# The evolution, complexity and diversity of models of long‐term forest dynamics

**DOI:** 10.1111/1365-2745.13989

**Published:** 2022-09-08

**Authors:** Harald Bugmann, Rupert Seidl

**Affiliations:** ^1^ Forest Ecology, Institute of Terrestrial Ecosystems, Department of Environmental Systems Science ETH Zurich Zürich Switzerland; ^2^ Ecosystem Dynamics and Forest Management Technical University of Munich Freising Germany; ^3^ Berchtesgaden National Park Berchtesgaden Germany

**Keywords:** Dynamic Global Vegetation Model, ecological modelling, forest gap model, forest landscape model, global change ecology, JABOWA, model design, model evolution

## Abstract

1. To assess the impacts of climate change on vegetation from stand to global scales, models of forest dynamics that include tree demography are needed. Such models are now available for 50 years, but the currently existing diversity of model formulations and its evolution over time are poorly documented. This hampers systematic assessments of structural uncertainties in model‐based studies.
2. We conducted a meta‐analysis of 28 models, focusing on models that were used in the past five years for climate change studies. We defined 52 model attributes in five groups (basic assumptions, growth, regeneration, mortality and soil moisture) and characterized each model according to these attributes. Analyses of model complexity and diversity included hierarchical cluster analysis and redundancy analysis.
3. Model complexity evolved considerably over the past 50 years. Increases in complexity were largest for growth processes, while complexity of modelled establishment processes increased only moderately. Model diversity was lowest at the global scale, and highest at the landscape scale. We identified five distinct clusters of models, ranging from very simple models to models where specific attribute groups are rendered in a complex manner and models that feature high complexity across all attributes.
4. Most models in use today are not balanced in the level of complexity with which they represent different processes. This is the result of different model purposes, but also reflects legacies in model code, modelers' preferences, and the ‘prevailing spirit of the epoch’. The lack of firm theories, laws and ‘first principles’ in ecology provides high degrees of freedom in model development, but also results in high responsibilities for model developers and the need for rigorous model evaluation.
5. *Synthesis*. The currently available model diversity is beneficial: convergence in simulations of structurally different models indicates robust projections, while convergence of similar models may convey a false sense of certainty. The existing model diversity—with the exception of global models—can be exploited for improved projections based on multiple models. We strongly recommend balanced further developments of forest models that should particularly focus on establishment and mortality processes, in order to provide robust information for decisions in ecosystem management and policymaking.

## Graphical abstract

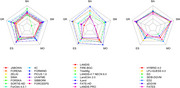

Editor's Choice

## 1 INTRODUCTION

Forests provide multiple ecosystem services from local to global scales that are crucial to humankind (FAO, 2020). However, anthropogenic climate change is jeopardizing the provisioning of multiple services in many parts of the globe (e.g. Lindner et al., 2010). Therefore, tools are needed to assess the impacts of climate change on forests, to evaluate their climate change mitigation potential, and to develop adaptive management strategies. Tree demography plays a key role in this regard. For example, tree mortality is pivotal for ecosystem biogeochemistry (Brienen et al., 2020; Bugmann & Bigler, 2011), and establishment processes are crucially determining ecosystem resilience after disturbance (Seidl & Tuner, 2022) as well as spatial shifts of species and ecosystems (Sharma et al., 2022). Thus, models that consider demographic processes in addition to growth are needed to study the long‐term interactions between forests and the climate system.

JABOWA, published 50 years ago by Botkin et al. (1972b), was the first individual‐based tree demography model for mixed‐species stands, aimed at capturing long‐term forest dynamics (here focused mainly on succession) along an elevational gradient covering 600 m in the Hubbard Brook Experimental Forest (Bormann & Likens, 1979). The success of JABOWA led to a proliferation of similar models—termed ‘forest gap models’—in the late 1970s and 1980s (Shugart, 1984). Forest landscape models (Mladenoff et al., 1996) and dynamic global vegetation models (Friend et al., 1997; Smith et al., 2001) were developed in the 1990s, with clear conceptual relations to forest gap models in terms of fundamental model assumptions. From the mid‐1980s onwards (e.g. Bugmann & Fischlin, 1994; Kienast, 1991; Solomon, 1986), forest gap models have increasingly been applied to study the impacts of climate change on ecosystem structure, composition and biogeochemistry. Half a century after their conception, forest gap models and models influenced by the early advances made by the gap modelling community are still in use for answering a wide range of fundamental and applied scientific questions (Maréchaux et al., 2021), including climate change impacts (for a brief review, cf. Bugmann, 2014). Subsequently, we refer to these models as ‘Models of Forest Dynamics’ (MFDs), acknowledging that there are other types of models (such as yield tables, forest growth models, species distribution models, Markov models, etc.) that we do not address here.

In contrast to physics, there are few fundamental theories, laws or ‘first principles’ in ecology based on which a forest model could be constructed. Thus, it remains challenging to mathematically capture tree demography, growth, competition and other key interactions in ecosystems in a way that allows for robust impact assessments under future no‐analog conditions (Williams & Jackson, 2007). The task of developing any model of long‐term forest dynamics is faced with a daunting number of degrees of freedom for the mathematical representation of individual processes (e.g. Huber et al., 2020), and this extends to processes that are perceived to be well‐understood, such as photosynthesis (Walker et al., 2021). This problem is even more acute when considering the feedbacks and interactions between individual processes within an ecosystem.

Our understanding of most ecological processes remains incomplete, and the mathematical representation of these processes in MFDs is uncertain. It is thus valuable to have different formulations available, either as alternatives within one model, or in the form of different models (i.e. using a different model architecture). If differently structured models provide sufficiently similar responses, for example to climate change scenarios (e.g. Sebald et al., 2021), our confidence that the simulated response is reflecting the system's true behavior—rather than being a model artefact—is increased. Thus, model comparisons and ensemble model simulations (e.g. Bugmann et al., 2019; Cramer et al., 2001; Fisher et al., 2018; Mahnken et al., 2022; Morales et al., 2005, Petter et al., 2020) are potentially of high value for increasing the robustness of projections and highlighting conditions under which our current systems understanding as formalized in models yields diverging results.

Yet, high agreement in model comparisons and ensemble simulations does not per se indicate low uncertainty. Model comparisons can yield high agreement under future scenarios not only if the models are ecologically robust but also if the key formulations underlying the models are sufficiently similar. In the most extreme case, comparing the projections of a group of equally ill‐designed models could result in the illusion of low model uncertainty. Also in science, we are confronted with ‘the prevailing spirit of the epoch’ (Baltensweiler & Fischlin, 1987), which is strongly shaping our activities. In the specific context of model development, this holds the danger of convergence in model formulations due to shared but not necessarily correct views. Thus, it is crucial to know how diverse the models being used actually are. In the past years, multiple review papers and comparisons of dynamic vegetation models from stand to global scales were published (e.g. Bugmann et al., 2019; Fisher et al., 2018; Larocque et al., 2016; Petter et al., 2020; Shifley et al., 2017; Shugart et al., 2018; Thurner et al., 2017; Yang et al., 2020), but they either covered only a small set of models, focused on selected processes, or remained qualitative in describing differences between models. To date, we lack a comprehensive approach to quantify the (dis)similarity in models that are used to address the same research question.

Fifty years provide ample time for a considerable evolution in models at the stand, landscape and global scale. Similar to a species that evolves based on changes in genome length and mutations in alleles, models evolve by the addition (or elimination) of features and changes in the formulation of individual model properties. This can lead to the convergence of approaches (e.g. when consensus model formulations replace previous, more diverse ones), or to diversification (e.g. when a broadening suite of scientific objectives results in more differentiated models). Given the original constraints on model complexity due to limited computational power (cf. Botkin et al., 1972a) and the strongly increasing ecological and ecophysiological knowledge over the past 50 years, we expect that the complexity of MFDs has increased considerably over time.

In this paper, we quantitatively evaluate the structure, complexity and diversity of MFDs, with a focus on models that are in use today to assess the impacts of climate change on forests. We compare the current models relative to JABOWA (Botkin et al., 1972b) as one of the foundational approaches to simulate forest dynamics, and ask how their complexity has changed over time. We furthermore quantify the current diversity in different model classes (stand, landscape, and global models) regarding their process formulations. Specifically, our research questions are as follows:

1. How have the complexity and diversity of MFDs changed over the last 50 years? Have developments at the stand, landscape, and global scales been different?

2. Can MFDs be clustered based on their attributes? If so, does this clustering reflect different fundamental aspects of models such as their scales of application (stand, landscape, global), or are other patterns of model (dis)similarity emerging?

3. Are MFDs currently being used for climate change impact assessments at different spatial scales balanced in their design with respect to the representation of key ecological processes such as the establishment, growth, and mortality of trees?

4. Are the basic assumptions underlying different MFDs (e.g. the entities being modelled and their spatial and temporal grain) pivotal for shaping their structure and complexity?

## 2 MATERIALS AND METHODS

### 2.1 Selection of models

For the present meta‐analysis, we did not aim to cover all individual‐based models that have been developed since the late 1960s; these were reviewed elegantly and comprehensively e.g. by Shugart (1984). Rather, we started from the first forest gap model, JABOWA, and focused our analysis on its numerous and widespread descendants to exemplarily illustrate the evolution of MFDs. Consequently, we used a two‐pronged strategy to identify the MFDs to be included in our analysis, as described below.

First, as a benchmark we selected models that we consider pivotal for forest modelling because they introduced new concepts or pioneered novel approaches. Starting from the first forest gap model, JABOWA (Botkin et al., 1972b), these models constitute distinct ‘founder events’ for the forest modelling community. This cohort includes the following models, ordered according to the date of their first publication.

In JABOWA (Botkin et al., 1972a, 1972b), the establishment, growth and mortality of individual trees as well as their competition for light are modelled based on simple ecological assumptions. JABOWA takes into account key environmental influences such as growing‐season temperature, drought, light availability, and crowding in dense stands. Trees interact with each other on small patches of land (typically, 100–1000 m^2^), and the behaviour of the forest as a whole is determined by averaging across multiple patches. This allows to consider both even‐aged as well as uneven‐aged stands. Within patches, horizontal heterogeneity (e.g. tree positions) is neglected, and there are no interactions between patches (e.g. via light availability or falling dead trees). The simplified structure of JABOWA enables the consideration of a wide range of species because requirements for parameter estimation are reasonably low. For more details on the basic structure of forest gap models, compare Shugart (1984).

FORENA (Solomon, 1986) was the first forest gap model applied along an extended climatic gradient, from the Canadian tundra to the subtropical mixed forests of the state of Georgia in the United States, spanning a range of mean annual temperature of *c*. 27°C. As such, it had to account for climatic influences on tree demography along this large gradient, which is a prerequisite for model applications under changing climatic conditions.

ZELIG (Smith & Urban, 1988) was the first forest gap model that considered horizontal interactions between the patches. It thus conceptually paved the way for forest landscape models, where horizontal spatial dynamics are essential.

FORSKA (Prentice et al., 1993) was designed to inject more biological realism into MFDs by basing most process formulations on physiological considerations. It thus provided the foundation for more sophisticated ecophysiological models that have a particular focus on the interactions between tree demography and biogeochemistry.

HYBRID (Friend et al., 1993) was the first model where a full‐fledged biogeochemical model (BIOME‐BGC) was coupled with elements of ‘conventional’ gap models to combine the strengths of both approaches in simulating tree demography and biogeochemistry. We acknowledge that at the same time, several other researchers were working on similar projects, including Martin (1992) and Bonan (1993), but HYBRID is the only model of this cohort that continues to be in use today. Also, HYBRID was a forerunner of what was to become the class of Dynamic Global Vegetation Models (DGVMs; see below).

SORTIE (Pacala et al., 1993) sought to escape (note the pun: *sortie* is a synonym of *foray*) from some fundamental constraints of JABOWA by abandoning the assumption of horizontal homogeneity of patches. Instead, it tracks individual tree positions explicitly, along with highly detailed calculations of incident radiation at the individual‐tree level. While earlier studies with forest gap models exist where within‐patch heterogeneity was explored (e.g. Busing & Clebsch, 1987), SORTIE was the first forest gap model to track individual tree positions.

LANDIS (Mladenoff et al., 1996) expanded gap model capabilities by including landscape‐scale processes such as seed dispersal, tree migration and an explicit representation of disturbances such as windthrow, insects and fire. We acknowledge that at the same time, Roberts (1996) and Keane et al. (1996) were working on similar ideas, albeit with a more limited scope on fire.

TreeMig (Lischke et al., 1998) was conceived as a landscape model but at the same time provided a bridge towards truly large‐scale applications of MFDs by pioneering model upscaling. Specifically, it replaced individual trees or tree cohorts by height classes and introduced a mathematical description of tree population dynamics using ordinary differential equations.

ED (Moorcroft et al., 2001) pursued the upscaling avenue further by using similar principles as in TreeMig, but applying them to a stand model of much higher complexity, particularly regarding the representation of ecophysiological processes. This enabled global applications of MFDs.

LPJ‐GUESS (Smith et al., 2001) achieved global‐scale applicability by further developing the principles underlying FORSKA, particularly invoking principles of ecological optimization. This resulted in a considerable simplification of computational demand while maintaining mechanistic representations of ecophysiological processes.

Second, we screened the recent (defined here as 2016–2021) literature via WebOfScience for applications of models to study the impacts of climate change on forest dynamics in mid‐November 2021. We identified 400+ entries and scrutinized these by hand, resulting in the selection of nine stand‐scale models, eight landscape‐scale models, and seven DGVMs (Table 1). Besides a focus on climate change, the models had to include processes of tree demography (i.e. at minimum tree establishment and mortality) with a reasonable level of detail. For example, models that just assume a turnover rate of biomass, rather than considering mortality as a demographic process, were excluded. Because some of the models that have recently been used in climate change assessments are among the ten founder models (i.e. ED, SORTIE, TreeMig, HYBRID, LPJ‐GUESS), and because some models have been used at both the stand and global scales (ED, ED2), the final set for the analysis comprised 28 unique models (Table 1). Five of the founder models (Table 1) are not in use any more today (i.e. JABOWA, FORENA, ZELIG, FORSKA and LANDIS). They are subsequently referred to as ‘legacy models’.

**TABLE 1:** Models included in the analysis, listed chronologically according to the date of their first publication. ‘Founder’ models (see the text for details) are printed in *italics*. In total, 28 unique models were analysed

Model	First publication	Recent^a^ climate change application
(a) Stand models		
*JABOWA*	Botkin et al. (1972b)	—
*FORENA*	Solomon (1986)	—
*ZELIG*	Smith and Urban (1988)	—
SIMA	Kellomäki et al. (1992)	Alrahahleh et al. (2018)
*FORSKA*	Prentice et al. (1993)	—
*SORTIE(‐ND)*	Pacala et al. (1993)	Moran et al. (2021)
ForClim	Bugmann (1996)	Huber et al. (2021)
4C	Bugmann et al. (1997)	Gutsch et al. (2018)
FORMIND	Köhler and Huth (1998)	Hiltner et al. (2021)
PICUS	Lexer and Hönninger (2001)	Boulanger et al. (2022)
UVAFME	Shuman et al. (2015)	Foster et al. (2019)
SIBBORK	Brazhnik and Shugart (2016)	Brazhnik et al. (2017)
ForCEEPS	Morin et al. (2021)	Morin et al. (2021)
(b) Landscape models		
*LANDIS*	Mladenoff et al. (1996)	—
Fire‐BGC	Keane et al. (1996)	Keane et al. (2019)
*TreeMig*	Lischke et al. (1998)	Scherrer et al. (2020)
LANDIS‐II	Scheller and Mladenoff (2004)	Olson et al. (2021)
LandClim	Schumacher et al. (2004)	Sebald et al. (2021)
iLand	Seidl et al. (2012)	Sebald et al. (2021)
FATE‐HD	Boulangeat et al. (2014)	Barros et al. (2018)
LANDIS PRO	Wang et al. (2015)^b^	Duan et al. (2021)
(c) Dynamic Global Vegetation Models		
*HYBRID*	Friend et al. (1993)	Thurner et al. (2017)
*LPJ‐GUESS*	Smith et al. (2001)	Schurgers et al. (2018)
*ED*	Moorcroft et al. (2001)	Ma et al. (2021)
SEIB‐DGVM	Sato et al. (2007)	Wu et al. (2019)
ED2	Medvigy et al. (2009)	Longo et al. (2018)
aDGVM	Scheiter and Higgins (2009)	Martens et al. (2021)
FATES	Fisher et al. (2015)	Holm et al. (2020)

^a^ Defined as published in the period 2016–2021.

^b^ As coupled to the LINKAGES v3.0 model.

### 2.2 Selection of model attributes (‘genes’) and their expression (‘alleles’)

We used a subjective and iterative approach to define model attributes for comparing the 28 models. Specifically, we distinguished between (1) basic assumptions (BA), (2) growth processes (GR); (3) establishment processes (ES), (4) mortality processes (MO) and (5) soil moisture processes (SM). The latter were included because at least *some* consideration of the water balance is needed to assess climate change impacts. Overall, we identified 52 relevant attributes in these five categories (Table 2). For each attribute, two to six levels of expression were defined; they are described in detail in Supplementary Material 1. To rephrase in terms of ‘model genomes’: The 52 attributes (‘genes’) feature a sum of 178 expressions (‘alleles’) and the potential for a total of $\prod_{i=1}^{52}n_{i}$ ≈ 6.7 × 10^30^ unique ‘genomes’, where *n*_*i*_ is the number of expressions of attribute *i*.

**TABLE 2:** Attributes considered in the analysis of 28 models of forest dynamics (cf. Table 1). The levels of expression of the attributes are described in Supplementary Material 1. Numbers in parentheses indicate the number of attributes in each category

Basic assumptions (8)	Growth (21)	Establishment 13)	Mortality (7)	Soil moisture (3)
Horizontal grain	Central state variable(s)	Approach	Background (BG) mortality: level	Vertical resolution
Horiz. structure within patches	Time step for tree geometry	Establishment probability	BG mortality formulation	Temporal resolution
Interactions between patches	Time step for productivity	Number of established trees	Stress‐related mortality	Drought
Vertical grain	Approach to model growth	Ingrowth threshold	Disturbance mortality	
Vertical extent of crowns	Allocation	Environmental influences	Windthrow	
Grain of modeled entities	Height‐DBH ratio	Light	Bark beetles	
Life forms considered	Leaf area‐DBH ratio	Moisture	Fire	
Focus of application	Crown length	Temperature		
	Crown width	Frost		
	Crown transparency	Browsing		
	Light extinction across the canopy	Seed production		
	Light response	Dispersal		
	Environmental influences	Vegetative reproduction		
	Time step for env. influences			
	Temperature			
	Soil moisture			
	Nutrients			
	CO_2_			
	WUE			
	Crowding			
	Phenology			

For each model, the expression of each attribute (if present) was assessed based on published papers, technical model documentations, model descriptions available on web pages, and in some cases also the model's source code. We specifically aimed to characterize the version of the model that had been used in a recent climate change impact assessment (Table 1). The list of attributes and their expressions for each model was subsequently sent to the respective PI of each model for cross‐checks and corrections. For the legacy models (Table 1), this task was accomplished by the first author of this paper. We received feedback from all but two PIs. Based on this feedback, we calculated the average error of our initial characterization of the 28 models. On average, 4.2 out of 52 attributes per model had to be corrected based on the feedback of the PIs. This corresponds to an error rate of 8.1% with a median of 3 erroneously assigned attributes per model. Extrapolating this to the two models for which no feedback from the PIs was received, an error rate of 2 × 4.2/(28 × 52) = 0.6% remains in the entire dataset. We deem this error unlikely to affect the outcome of our analyses.

All attribute expressions were converted to a numerical scale with equal distances between attribute expressions, ranked by increasing complexity. The numerical expressions were scaled to an average of zero and a standard deviation of one for each attribute. This approach is an established method in clinical psychological research for quantifying the results of qualitative surveys (cf. Kline, 2015; Schweizer & DiStefano, 2016): our 52 model attributes are equivalent to the questions of a structured survey (test), and the expressions of the attributes are equivalent to the standardized answers of the surveyed persons, which in our case are the 28 models. To assess the sensitivity of the method, we evaluated varying distances between attribute expressions and found the results of the analyses to be robust to such variations (data not shown).

### 2.3 Analysis of the models

First, we analysed changes in model complexity over time. We did this based on the year of first publication (cf. Table 1) with two exceptions: The FIRE‐BGC and 4C models have undergone strong conceptual and structural development since their first publication. Therefore, for these models we used the year when the currently applied version was published, that is Keane et al. (2011) and Lasch‐Born et al. (2020), respectively.

We specifically focused on (1) the number of attributes that were considered (‘genome length’), and (2) the average complexity in the five attribute groups BA, GR, ES, MO, and SM. Furthermore, radar plots were drawn to visualize the complexity of each model for these five attribute groups and the diversity within the classes of stand, landscape and global models.

Second, numerical distances between the models were calculated using multiple distance measures including Euclidean, Manhattan, Canberra and Minkowski. Results were generally found to differ little, and the Canberra distance metric produced the ecologically most plausible results. Similarly, multiple clustering algorithms were tested, including Ward, Ward.2, single, complete, and average. Also here, the results differed little. Furthermore, a range of multivariate analysis techniques such as metric multi‐dimensional scaling and k‐means clustering were evaluated, yielding similar results compared to those from hierarchical clustering. These analyses indicate that our findings are robust to different techniques being applied. Here, we report the results for hierarchical clustering with the complete method based on Canberra distances. The clustering was done for the models as a whole, and separately for the attribute groups GR, ES, and MO.

Third, heat maps were drawn to visualize the attribute space, both unclustered and clustered, using hierarchical clustering based on the methods described above. The optimum number of clusters was five, being determined using 22 indices and the majority rule.

Lastly, to evaluate whether the basic assumptions underlying the models influence their structure and complexity, we conducted a redundancy analysis (RDA) using the set of BA attributes, the model class (stand, landscape, global) and the time of first model publication (Table 1) as explanatory variables for the expressions of the other attributes.

All analyses were conducted in the statistical software R version 4.1.2 (R Core Team, 2021). For radar plots, package fmsb (Nakazawa, 2021) was used. Complex hulls were drawn using package grdevices. Distance matrices between the models, hierarchical clustering and metric multi‐dimensional scaling (MDS) were calculated using the stats package. The optimum number of clusters was determined using package nbclust (Charrad et al., 2014). k‐means clustering was performed using the factoextra package (Kassambara & Mundt, 2020). Heat maps were drawn using package pheatmap (Kolde, 2019), and redundancy analysis was performed using package vegan (Oksanen et al., 2020).

## 3 RESULTS

### 3.1 Temporal evolution of model complexity and diversity

**FIGURE 1:**
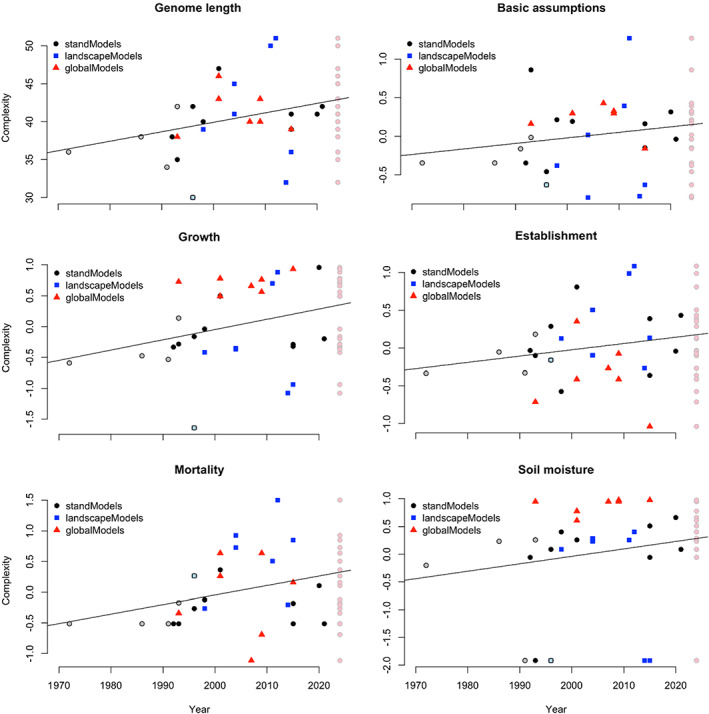
Temporal development of genome length (i.e. number of attributes considered by a model) and the complexity of the basic assumptions, growth, establishment, mortality, and soil moisture attributes. Legacy models (no longer in use today) are shown in light colours. At the right of every panel, the models still in use today are shown with light red circles to illustrate current model diversity. Linear trends are provided merely for better visualization; they are not meant to be statistically meaningful.

The number of attributes being modelled (i.e. ‘genome length’) and the complexity of the formulations being used for each attribute increased over time. Increases were strongest for growth attributes and weakest for the complexity of basic assumptions and establishment attributes (Figure 1). Genome length increased particularly due to the recent development of complex landscape and global models. Global models also contributed strongly to an increase in the complexity of basic assumptions and even more so of growth attributes. In contrast, these models tend to feature a comparatively simple representation of establishment attributes. The complexity of mortality attributes increased particularly due to the majority of the more recently developed landscape models. Finally, also the complexity of soil moisture attributes increased, particularly due to the development of global models. The models currently being used for climate change impact assessments are highly diverse in their overall complexity. Diversity is highest for establishment and mortality attributes, and lowest for soil moisture attributes.

### 3.2 Differences between stand, landscape, and global models

**FIGURE 2:**
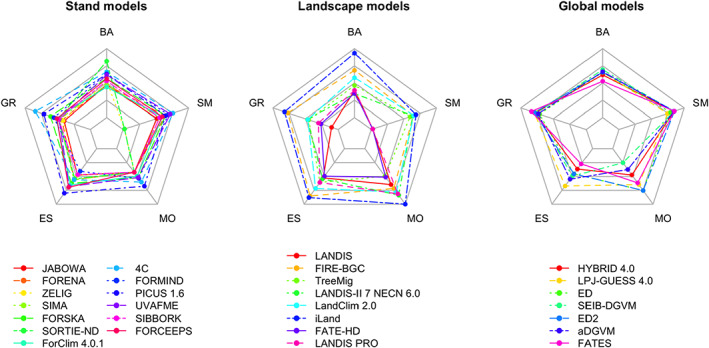
Average values for the attributes of the 28 models by attribute group [basic assumptions (BA); growth (GR), establishment (ES), mortality (MO) and soil moisture (SM) processes], and scale of model application (model class).

When analysing model complexity and diversity for stand, landscape and global models separately (Figure 2), distinctly different patterns emerged for the three model classes (for details see Table S1). Global models feature the lowest diversity across all five attribute groups. They are characterized by generally high complexity and low diversity in basic assumptions as well as growth and soil moisture attributes. At the other end of the spectrum, landscape models were found to be the most diverse class of models in all five attribute groups. Their average complexity is highest for establishment and mortality attributes, but lowest with regard to growth and soil moisture attributes. Stand models feature intermediate diversity for all five attribute groups. We also found them to have intermediate complexity for all attribute groups except for mortality, where stand models are on average applying the simplest model formulations. Part of the differences in diversity between stand models and the other two classes might be explained by the larger number of stand models included in our analysis (*n* = 13). However, an imbalanced sample cannot explain the strong differences in diversity between landscape and global models, as nearly the same number of models was analysed for these two classes (*n* = 8 and 7, respectively).

**FIGURE 3:**
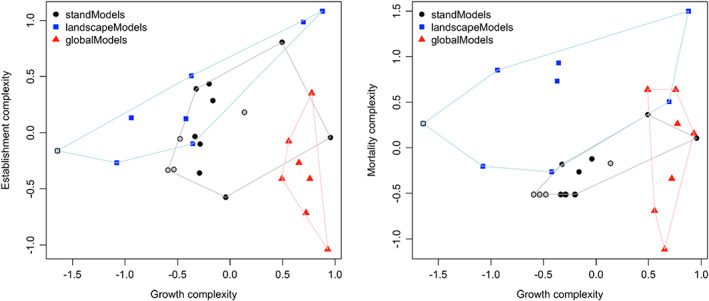
Niche differentiation of stand, landscape and global models with respect to growth (GR), establishment (ES) and mortality (MO) attributes. Legacy models (no longer in use today) are shown in light colours.

A direct comparison of the complexity of growth, establishment and mortality attributes by model class (Figure 3) revealed a clear ‘niche differentiation’: global models excel in the complexity of growth formulations, whereas landscape models feature the most complex formulations of establishment and mortality attributes, with stand models ranking in between the other two classes. The legacy models tend to have lower complexity compared to models that are still in use today.

### 3.3 Emerging model clusters

**FIGURE 4:**
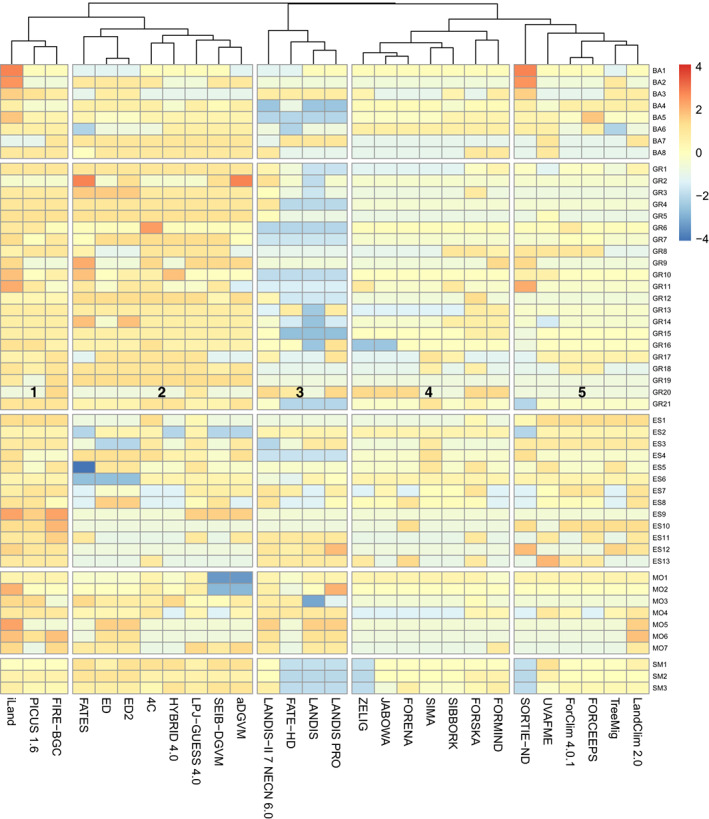
Hierarchical clustering and heat map of 28 forest simulation models based on 52 attributes. The clustering uses Canberra distances and the ‘complete’ clustering method. Blue and red colours indicate low and high complexity for each attribute, respectively. Gaps between columns delineate five main clusters, which are identified by numbers in the middle of the heat map for easier reference in the text. Gaps between rows indicate the boundaries between the five attribute groups (from top to bottom: basic assumptions, growth, establishment, mortality and soil moisture processes). Numbers to the right of the rows indicate the respective attribute number (cf. Supplementary Material 1).

When analysing model complexity at the level of individual MFDs using heat maps, no distinct patterns discerning the three model classes are visible (cf. Figure S1). Thus, the spatial domain of a model (stand, landscape, globe) is a weak predictor of its structure and complexity. When the models are clustered regardless of their a priori designation to a class, however, clear patterns of similarity emerge (Figure 4; cf. Figure S2 showing just the clustering and Figure S3 showing a similarity matrix).

Overall, five distinct model clusters emerged (Figure 4). We start their analysis with the fourth cluster, which includes stand models that have remained relatively similar to the foundational model JABOWA. Note that this cluster includes four of the five legacy models in the set. Also included in this cluster yet set apart clearly from the rest are FORSKA and FORMIND, which feature considerably higher complexity in several attributes, particularly with regard to tree growth.

The fifth cluster is linked closely to the fourth cluster. It contains four stand models that feature higher complexity (Figure 4) particularly with respect to growth and establishment attributes compared to the models in the fourth cluster. SORTIE is part of the fifth cluster as well, but it is separated distinctly from the other models, reflecting the fact that its assumptions and structure deviate strongly from those of the other models in this cluster. It is further remarkable that two landscape models, TreeMig and LandClim, are part of this cluster. Both models were derived from the stand model ForClim, and in spite of added spatial features the remainder of their structure is broadly similar to that of the stand models in the fifth cluster.

The third cluster is clearly separated from the others and contains four landscape‐scale models (Figure 4). They feature lower complexity with respect to basic assumptions, growth, establishment, and soil moisture attributes compared to all other clusters. However, LANDIS‐II is clearly different from the other three models in this cluster, as it has more complex formulations with regard to a number of attributes. Most models in this cluster have highly complex mortality formulations, which is due to the spatially explicit nature of landscape models and their focus on disturbance processes.

The second cluster unites the seven global models of the set and includes the stand model 4C (Figure 4). This assignment of 4C to the global model cluster is robust regardless of the distance metric or clustering method used (results not shown). The models of this cluster share highly complex basic assumptions as well as growth and soil moisture attributes. The diversity of attribute expressions is particularly low in this cluster.

Lastly, the first cluster brings together two models developed by scientists who worked together for an extensive period of time, that is the stand model PICUS (PI Lexer) and the landscape model iLand (PI Seidl), along with the landscape model FIRE‐BGC. These three models always formed a cluster of their own regardless of the clustering method, reflecting the fact that iLand (Seidl et al., 2012) was partly inspired by both PICUS (Lexer & Hönninger, 2001; Seidl et al., 2008) and FIRE‐BGC (Keane et al., 2011). This cluster features high complexity across all attribute groups.

### 3.4 Relationships at the level of ecological attribute groups

**FIGURE 5:**
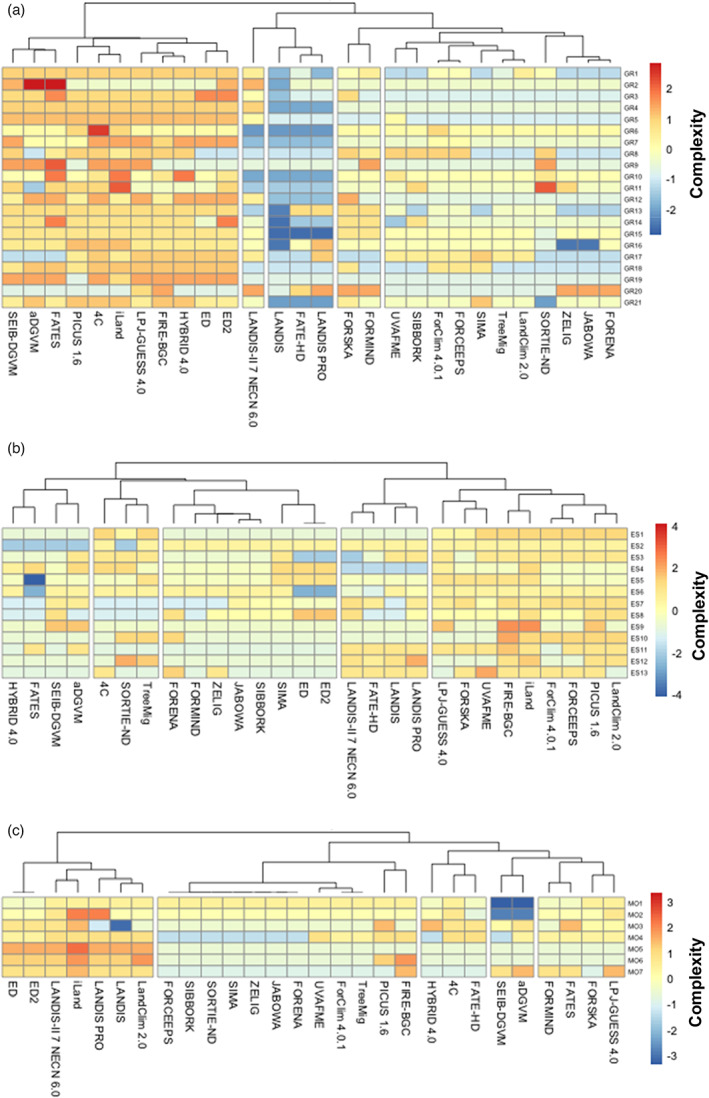
Hierarchical clustering and heat map of 28 forest simulation models for the demographic attribute groups (a) growth (GR), (b) establishment (ES) and (c) mortality (MO). The clustering uses Canberra distances and the ‘complete’ clustering method. Numbers to the right of the rows indicate the respective attribute number (cf. Supplementary Material 1).

Clustering at the level of the three fundamental processes of forest dynamics, that is, growth, establishment and mortality, reinforces and sharpens the interpretations made above. When looking at growth attributes (Figure 5a), FORSKA and FORMIND are separated from the bulk of the other stand models; LANDIS‐II is separated from the three low‐complexity landscape models; and PICUS, iLand and FIRE‐BGC are found in the same cluster as all global models (and 4C), being characterized by high complexity.

Regarding establishment attributes (Figure 5b), models of low complexity (clusters 1–3) from all three model classes are separated from those of intermediate (cluster 4, exclusively landscape models) and high complexity (cluster 5, no clear model class).

Regarding mortality attributes (Figure 5c), five landscape models of higher complexity are grouped with ED/ED2 in the first cluster, whereas the other two landscape models (which feature a lower number of spatially explicit processes) are grouped in the second cluster with most stand models. In the third cluster, models with intermediate to relatively high complexity regarding the formulation of ‘background’ (attributes #43 & 44) and ‘stress‐related’ (#45) mortality but low complexity regarding spatially explicit processes (#47–49) are found. The fourth cluster contains two models that do not contain a ‘background’ mortality rate at all (#43 & 44), while both consider fire disturbance (#49). The grouping in the fifth cluster is difficult to interpret.

### 3.5 Relationship between basic assumptions and model structure

**FIGURE 6:**
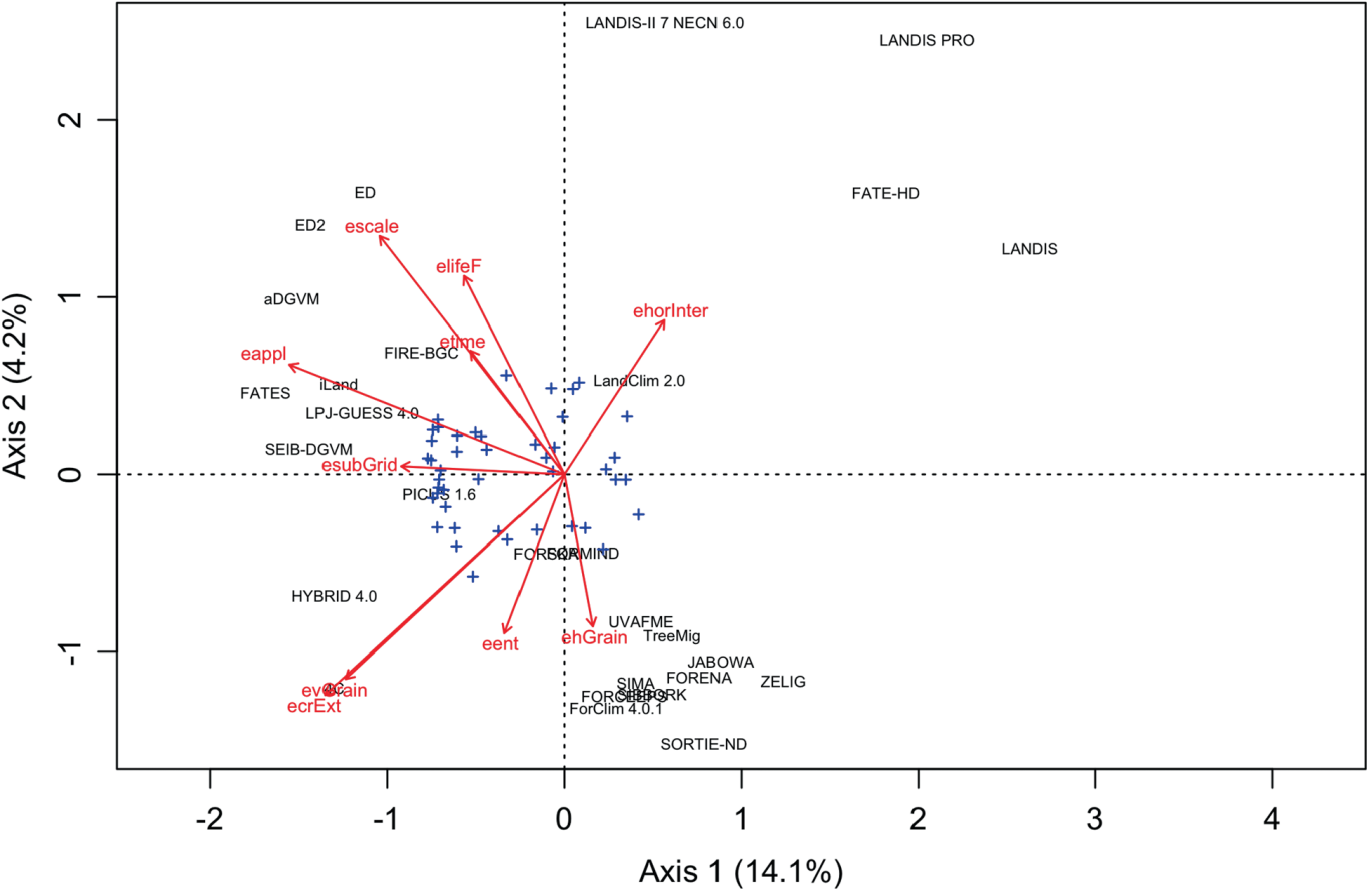
Redundancy Analysis (RDA) to explain the expression of attributes of the 28 models as a function of the eight basic assumptions (BA), scale of application and time of first publication (arrows). The blue crosses represent the 52 attributes. ehGrain:Horizontal grain (#1) subGrid: Structure within patches (#2) ehorInter: Interactions between patches (#3) evGrain: Vertical grain (#4) ecrExt: Vertical extent of crowns (#5) eent: Grain of modelled entities (#6) elifeF: Life forms considered (#7) appl: ocus of application (#8) time: ear of first model publication escale: Scale of model application (stand, landscape, global).

The redundancy analysis (RDA) had an $R_{\mathrm{adj}}^{2}$ of 0.43. Yet, the constrained variance was only 80% larger than the unconstrained variance, suggesting a limited power of the eight basic assumptions along with model class and time of first publication for explaining model structural features. Still, the structure of six global models (top left in Figure 6; along with FIRE‐BGC and iLand) is closely related to their focus of application (structure, composition *and* biogeochemical cycling), their spatial extent (i.e. model class) and the life forms considered (i.e. all global models include a representation of the herbaceous understory). Conversely, the structure of five landscape models (top right in Figure 6) is closely related to the presence of horizontal interactions. Lastly, the structure of two models, HYBRID and 4C, is closely related to their vertical grain and the modelling of the vertical extent of tree crowns.

## 4 DISCUSSION

### 4.1 Temporal development of model complexity and diversity

The increasing complexity of MFDs over the past 50 years reflects enhanced ecological understanding. For example, what is now a standard photosynthesis model was formulated only in 1980 (Farquhar et al., 1980). Similarly, understanding plant carbon allocation was in its infancy in the 1970s (Webb, 1977) and remains a challenge even today (Hartmann et al., 2020; Merganicova et al., 2019). Furthermore, strongly increasing computational capacities (Waldrop, 2016) made it possible to include more complex process formulations in MFDs while maintaining or even lowering computing time.

The temporal development of growth and soil moisture complexity was largely driven by global models, as their original emphasis was on carbon exchange between the biosphere and the atmosphere (Bonan, 1991), which is intricately linked to the water cycle (Sellers et al., 1986), thus leading to complex formulations. These, however, can be simplified based on optimality theory (e.g. Harrison et al., 2021). At the landscape scale, growth processes are not the major driver of vegetation dynamics (Elkin et al., 2012), but demography and disturbances strongly determine landscape patterns. This partly explains the relatively simple growth formulations in many landscape models (e.g. FATE‐HD). However, their high simplicity raises questions regarding their suitability to assess climate change impacts comprehensively. Approaches as adopted in LANDIS PRO (Duan et al., 2021), where a modified forest gap model (LINKAGES v3.0; Dijak et al., 2017) was coupled with a landscape model, might present a way forward. However, we posit that it is more coherent and elegant to upgrade the growth process formulations within landscape models themselves (cf. Schumacher et al., 2004; Seidl et al., 2012).

The increase in complexity regarding mortality and establishment formulations over time is largely due to landscape model development. In these models, crucial processes such as seed production, dispersal and tree establishment must be considered, and spatially explicit disturbances that kill trees and allow for establishment of new trees play a pivotal role. Consequently, current landscape models have high potential for quantifying forest resistance and resilience to climate change (Albrich et al., 2020; Turner et al., 2022). The high level of detail in demographic processes in landscape models is contrasted by a low establishment complexity of many global models, which we view as a source of concern. For example, assessing the consequences of Amazon rainforest dieback (Boulton et al., 2017) depends not only on accurate modelling of tree mortality but also on capturing establishment processes following drought‐induced mortality. Ecophysiology alone is unlikely to be sufficient to capture the interactions between climate and forest dynamics.

Stand models have contributed least to the overall increase in MFD complexity over the past five decades. Yet, notable exceptions of complex stand level models are FORSKA, FORMIND, PICUS and particularly 4C. This limited increase is partly due to the fact that most of the relevant processes at the stand level were already included in JABOWA 50 years ago, albeit at a much lower level of complexity. The original JABOWA model was developed to capture vegetation properties along a 600 m elevation gradient, that is a range of *c*. 3°C in mean annual temperature, which was possible using a number of simplifying assumptions. For FORENA, this range expanded to 27°C (Solomon, 1986), and the simulation of climate change scenarios brought the challenge to represent the ecological impacts of no‐analog climate conditions. This required comprehensive formulations for the influence of climate in MFDs already 30+ years ago, although some of them were inflicted with conceptual problems (cf. Loehle & LeBlanc, 1996).

The evolution of process formulations in MFDs over the past 50 years was clearly influenced by major ecological developments. The importance to better understand the Earth's carbon balance in the context of climate change and the need to resolve the origin of the ‘missing sink’ (Gifford, 1994) profited from an improved understanding of tree physiology, and led to more elaborate models of tree growth (e.g. 4C, FORMIND). The emergence of new sub‐fields of ecology, such as landscape ecology (in the 1980s; Turner, 1989) and macroecology (in the 2000s; Gaston & Blackburn, 2000) fuelled the development of MFDs operating at the landscape and global scale. More recently, tree mortality has come into focus of model developers, not least because of reports of increasing tree mortality in many parts of the world (Kharuk et al., 2021; Parks & Abatzoglou, 2020; Senf et al., 2021), and expectations of pervasive shifts in these processes in a changing world (McDowell et al., 2020). The rather moderate increase in the complexity of regeneration modelling approaches reflects the fact that the drivers of tree regeneration remain difficult to grasp (Bugmann et al., 2019) and aligns with recent calls for a renewed focus on regeneration processes in forest ecology (Seidl & Tuner, 2022).

Overall, increasing the complexity of process formulations in MFDs was motivated by accommodating new process understanding and enhancing model accuracy. However, this does not imply that higher complexity always leads to better projections. Overly complex models tend to be prone to reduced transparency, robustness and predictive power (Franklin et al., 2020). After all, the objective of modelling is to simplify a complex reality (cf. Pace, 2003), and the objective of any given model application dictates the necessary model structure. For example, models aiming to reproduce hourly or daily patterns of net ecosystem productivity over a couple of years (e.g. Harrison et al., 2021) need much higher temporal resolution in simulated growth and soil processes than models aiming to project annual tree growth over decades to centuries (e.g. Irauschek et al., 2021). Thus, the need for model complexity has to be substantiated relative to model purpose (cf. Albrich et al., 2020), and general statements on what processes need to be included in an MFD and at what level of complexity are futile.

Lastly, we recommend that model development should always proceed in a way that added complexity is implemented as optional features, that is that previous model versions remain retrievable. This avoids the tendency towards ‘baroque’ models (Prentice et al., 2015) and allows for tailoring the complexity of models to the research question at hand (Fisher & Koven, 2020).

### 4.2 Differences between stand, landscape, and global models

While the overall set of models considered here is highly diverse in both the number of processes considered and their complexity, this diversity varies strongly between model classes (stand, landscape and global) and attribute groups.

Stand models feature intermediate complexity in most attribute groups, and they are also of intermediate diversity compared to the other two classes. Consequently, the structure and complexity of stand models have not converged even 50 years after their first appearance. In fact, three paradigms are underlying the current diversity of stand models: (i) models that remain quite similar to early model formulations (e.g. SIMA, SIKBBORK); (ii) models that are still simple but have a substantially enhanced model structure geared towards wide applicability and robustness (e.g. ForClim, UVAFME); and (iii) highly detailed process‐based forest gap models (e.g. FORMIND, 4C). This provides the opportunity for comparative simulation studies to assess the robustness of simulation results under climate change by using models from more than one of the three paradigms.

The very high diversity of model formulations at the landscape scale partly reflects the fact that some of these models (e.g. FATE‐HD, to some extent also LANDIS) are inspired by state‐and‐transition models (e.g. Noble & Gitay, 1996) and thus build on simple schemes particularly with regard to growth attributes. Other forest landscape models have their roots with ‘classical’ gap models (e.g. LandClim, TreeMig). Still others draw heavily on physiology‐oriented stand models (e.g. PICUS) or biogeochemistry models (e.g. BIOME‐BGC), resulting in the most complex landscape models in our set, that is FIRE‐BGC and iLand. This diversity in landscape models should be explored more explicitly in comparative studies to understand the robustness of landscape level projections (e.g. Sebald et al., 2021). Yet, the first formal forest landscape model comparison has emerged only recently (Petter et al., 2020).

The global‐scale models were the least diverse in our set across all attribute groups. This reflects two phenomena.

On the one hand, a major original objective for these models was to simulate global NPP (e.g. Haxeltine & Prentice, 1996). NPP arises from the balance of photosynthesis and autotrophic respiration, which are understood reasonably well at the physiological level. This led to the inclusion of similar formulations for carbon (C) dynamics and plant hydraulics across multiple global models. Examples are the paradigmatic Collatz et al. (1991) photosynthesis model and the model of stomatal conductance by Leuning (1995). These similarities raise the expectation that simulated outcomes of, for example, global NPP are similar, which paradoxically is not the case (Prentice et al., 2015).

On the other hand, the focus on C exchange led to low complexity regarding demographic processes, such that some relevant processes are not simulated at all in some global models (e.g. aDGVM includes only one single mortality process, which is stress‐related mortality). Therefore, an upgrade of global models with regard to tree demographic processes is needed to increase their utility for assessing future forest trajectories (cf. Brienen et al., 2020; Friend et al., 2014). Recent developments, however, indicate that the focus on photosynthesis and plant hydraulics continues (Harrison et al., 2021). We nonetheless agree wholeheartedly with these authors that ‘*model development should be refocused on the critical analysis and evaluation of core process representations, and new processes added only if evidence unambiguously shows that they are required*’. Yet, our interpretation of ‘core processes’ may differ.

### 4.3 Emerging model clusters

The cluster analysis confirmed that model class (stand, landscape, global) is only a weak predictor of model properties. An exception are global models, which were all part of the same cluster. The stand model 4C was also part of this cluster, as it is highly complex regarding its growth and soil moisture attributes, but considerably less so with respect to establishment and mortality attributes.

The fact that the landscape models were distributed across three of the five clusters (Figure 4) reflects their diverse ancestry. Specifically, the placement of LandClim and TreeMig in the same cluster as ForClim reflects the fact that these models are closely related to ForClim (Bugmann, 1996). Similarly, the placement of iLand with Fire‐BGC and PICUS reflects the influence of the latter in the development of the former.

The cluster that contains JABOWA and three other legacy stand models is characterized on the one hand by models that have remained very similar to the ancestor JABOWA, and on the other hand three models that differ considerably from the earlier approach, i.e. SORTIE and two models that are characterized by much higher mechanistic detail (FORSKA, FORMIND), which may be surprising as their mathematical structure is rather different indeed. It is noteworthy that with the exception of FORSKA (a legacy model) and FORMID, the rather traditional formulations used in various parts of the models of this cluster (e.g. degree‐day parabola in SIMA—cf. Loehle, 1996, or the number of ‘dry days’ as a drought indicator in SIBBORK—cf. Fischlin et al., 1995) raise questions how reliable projections from these models will be in a changing climate. The other cluster that is dominated by stand models (cluster 5 in Figure 4) is characterized by considerable advances in this regard, while remaining simple in terms of environmental influences on tree demography (e.g. degree‐day asymptote and ratio of soil moisture supply to demand as a drought index in ForClim) and the representation of tree competition.

Again, modelling objectives inevitably determine appropriate model structure. Consequently, there is ample scope for models that are not highly ‘mechanistic’ but based on simpler representations of tree growth and demography. The fact that there is a large body of knowledge about a certain process (e.g. photosynthesis) does not imply that this process has to be included with a high level of complexity in all models. High complexity is only warranted if it is crucial for accurately and robustly achieving study objectives.

### 4.4 Relationships at the level of ecological attribute groups

The most striking insight from the cluster analysis at the level of attribute groups (Figure 5) compared to the cluster analysis at the level of full model ‘genomes’ was that there are strong differences between these two analyses (Figure 4). This shows that many models are not balanced in their complexity across different attribute groups. This is partly due to differences in importance of processes for certain applications (e.g. establishment and mortality are more important in landscape and partly in global models because disturbances are important at these scales; or global models need to simulate a closed carbon budget to infer the climate change mitigation function of global forests). However, these differences are in part also due to legacies (cf. Harrison et al., 2021), modelers' preferences, and perhaps also ‘the prevailing spirit of the epoch’ (Baltensweiler & Fischlin, 1987). For example, within the biogeochemical modelling community, the use of a specific form of the Farquhar‐Caemmerer model of leaf photosynthesis is usually taken for granted, although considerable uncertainty exists around this formulation (cf. Walker et al., 2021). Similarly, funding agencies sometimes are trapped in mainstream thinking regarding what should be funded, thus narrowing the scope of possible model development trajectories.

Specifically, out of the 23 models still in use today, only one was always found in a cluster that was ranked similarly regarding its complexity in the three attribute groups (i.e. iLand, which was designed with this goal, cf. Seidl et al., 2012). While a few other models were found in clusters of similar complexity (SORTIE, TreeMig, SIMA, SIBBORK and LPJ‐GUESS), the majority of models (17) were in clusters that indicate highly different complexity with regard to growth, mortality and regeneration processes.

When screening model descriptions in the context of this meta‐analysis, it was notable how some models excel in the detail that is devoted to physiological growth processes, covering many dozen equations and many pages of documentation. However, equally important aspects such as establishment and mortality often appear like an afterthought, with a few lines of text and code, in comparison. This is in stark contrast to the availability of potentially useful research results, syntheses, and recommendations (e.g. Adams et al., 2017; Cailleret et al., 2017; Sharma et al., 2022; Thrippleton et al., 2021) and calls for a more balanced consideration of ecological processes in model development.

### 4.5 Relationship between basic assumptions and model structure

The fact that the basic assumptions underlying the models were only weak predictors of their structural attributes underlines the scarcity of theories, laws and ‘first principles’ in the ecological sciences. One possibility is that the attributes to capture basic assumptions were not chosen appropriately. Alternatively, they may not provide rigorous constraints on further model attributes, thus leaving many decisions of model formulation to the developers. We lean towards the latter interpretation, as it corresponds to our own multi‐decadal experience in developing MFDs. This underlines the responsibility that lies on the shoulders of modellers. It is important to keep in mind that modelling is a dedicated scientific activity with strong fundaments in the philosophy of science (Winsberg, 2010); it should be performed rigorously and, ideally, based on in‐depth formal training (Ewing et al., 2003; Seidl, 2017). For example, conceptual models, mathematical models and computer code (simulation models) are different entities. It is pivotal to clearly recognize the role and importance of each of these steps in modelling. In other words: modelling should not be mistaken as the activity of adding code to existing simulation models.

### 4.6 Methodological considerations

Our analysis revealed multiple patterns of complexity across different model classes and attribute groups; all of these patterns were robust to variations in analysis methods. Therefore, we are confident that our findings are not artefacts resulting from erroneous assumptions or inappropriate data analysis. Specifically, we explored a number of different approaches for statistical analysis but never obtained substantially different results.

Yet, it is clear that the definition of the 52 attributes and their expressions is inherently subjective. We consciously approached this task by iteratively developing attributes and testing them on the four models that we are intimately familiar with because we either led their development or were involved in it (PICUS, iLand, ForClim and LandClim). A second test case of the iterative development of the attributes were the legacy models, which we also know quite well. The attribute expressions were further refined and expanded in the assessment of the other 19 models of the set. Importantly, our assessment of model expressions for 17 out of these 19 models was cross‐checked by the respective PIs, such that no errors should remain in 26 of the 28 models. Also, in the vast majority of cases the PIs found our attributes clear and well‐defined, which suggests that the system we developed (cf. Supplementary Material 1) is appropriate and useful. This also suggests that further models can be added to the model characterization presented here, serving as a consistent and comprehensive framework for cataloguing MFDs.

We also acknowledge that using the year of the first publication of a model (with the exception of FIRE‐BGC and 4C, as noted in the Materials and Methods section) for characterizing model complexity over time may induce a bias, since many models are undergoing continuous development. However, the conceptual basis, fundamental assumptions and structure of a model, that is, the main elements captured in our attributes, normally are rather stable. Thus, while we know that some of the scatter that we found in the development of structural diversity over time is due to an imprecise estimate of the actual introduction of any given approach, this is unlikely to influence the overall pattern reported here.

Lastly, it is clear that our analysis necessarily remains coarse at the level of individual attributes, only considering between two and six different expressions per attribute. Specifically, we did not aim to classify models at the grain of individual mathematical equations, because this would not have been tractable in both the assessment and analysis phases of this work. Nonetheless, given the relatively high number of attributes even our coarse characterization of attributes resulted in the potential for 6.7 × 10^30^ unique descriptions of models, and our analyses demonstrating the considerable diversity and variation between models underlines the utility of our approach.

## 5 CONCLUSIONS

Over the past 50 years, the complexity of models of forest dynamics has increased substantially. This partly reflects enhanced ecological knowledge and strongly increasing computing power, but partly also the desire to develop models that more realistically represent natural processes. Whether this increased complexity is warranted must be judged based on the objective of individual model applications; there are no general rules. Model diversity is generally high—we did not find evidence towards a convergence in model formulations, which we view as being beneficial. However, model diversity has developed quite differently for different model classes. The very low diversity of some formulations in global models may be detrimental, and diversification should be sought to improve the robustness of multi‐model assessments at the global scale. Yet, we note that for some processes such as soil moisture dynamics, where physical principles are fairly well established, low diversity may not necessarily be a problem.

While global models were clustered together in our analysis, landscape models fell into three different clusters, reflecting different underlying paradigms of model formulation and high model diversity. Stand models fell broadly into two clusters, with less complex models that are very closely related to JABOWA, and more developed yet still simple models in another cluster. We propose that the model diversity documented here should be harnessed more deliberately in multi‐model assessments and ensemble simulations towards more robust forest projections. Specifically, we argue that models in multi‐model assessments should be selected to cover the variety of available approaches. The cluster analysis presented here can help inform such studies. Furthermore, differences in simulated outcomes in multi‐model assessments could be related to the (dis)similarity of individual models identified here (cf. Figure S3) in order to contextualize remaining uncertainties in simulation‐based studies.

Most models in the set were not well balanced in their level of complexity across different attribute groups. On the one hand, this reflects the different objectives of the different model classes, and is thus an inherent aspect of specific models (i.e. a purpose‐driven simplification of a complex reality). On the other hand, this imbalance reveals implicit paradigms underlying the currently available models, such as the strong focus on growth processes in global models. Where such a focus comes at the cost of low resolution in other important processes such as mortality and establishment, biased projections under climate change are the likely outcome. We thus strongly welcome the ongoing change in perspectives in this regard, with an increased focus of the global modelling community on demographic processes (e.g. Fisher et al., 2018; Pugh et al., 2019).

Lastly, our analysis shows that there are few constraints on the structure and complexity of MFDs arising from their basic assumptions, which continues to make the development of a computer model of forest growth challenging, even 50 years after the initial gap model JABOWA was published. Yet, the resulting diversity of modelling approaches is an asset in the context of multi‐model applications, enabling the assessment of the robustness of projections under climate change. This is a standard in the global modelling community already, and should be adopted more widely also in the stand and landscape modelling communities. The large diversity of models also makes it imperative that they are evaluated thoroughly against independent data before they are applied in the context of decision support, scrutinizing the ability of any given model formulation to represent the dynamics of the respective study system.

## AUTHOR CONTRIBUTIONS

Harald Bugman and Rupert Seidl conceived the ideas and designed the methodology; Harald Bugman collected and analysed the data and led the writing of the manuscript; Harald Bugman and Rupert Seidl revised and finalized the manuscript.

## CONFLICT OF INTEREST

The authors declare no conflict of interest.

### PEER REVIEW

The peer review history for this article is available at https://publons.com/publon/10.1111/1365‐2745.13989.

## Supporting information


Appendix S1

## Data Availability

All scripts and data used in this paper are available from the following *Dryad Digital Repository* https://doi.org/10.5061/dryad.qfttdz0m2 (Bugmann & Seidl, 2022).
