# Supplementary material for: The evolution, complexity and diversity of models of long‐term forest dynamics
Source: J Ecol. 2022 Sep 8;110(10):2288–307. doi: 10.1111/1365-2745.13989 (PMC9826524; doi:10.1111/1365-2745.13989)
Supplement: Supplementary file 1 — Appendix S1 [file JEC-110-2288-s001.zip › JEC_13989_Genealogy Paper SM1 (atttributes).docx]

# Supplementary Materials

## SM 1: Definition of model attributes and levels of expression

### a) Basic assumptions (BA)

1. **Horizontal grain –** The resolution of the model in the horizontal dimension:
   (i) stands (>1000 m^2^)
   (ii) patches of 100-1000 m^2^ size, conforming to the original assumption in forest gap models
   (iii) patches smaller than 100 m^2^
   (iv) individual trees with x,y coordinates.
2. **Horizontal structure within patches –** Is there any horizontal structure within the patches:
   (i) horizontally homogeneous
   (ii) taking into account sub-grid scale heterogeneity by some means (e.g., sub-patches)
   (iii) individual trees are resolved.
3. **Interactions between patches –** Are there horizontal interactions between the patches other than disturbances (the latter are handled under “Mortality”, cf. below):
   (i) NA (no, not modeled = not available)
   (ii) Light availability
   (iii) Seed dispersal
   (iv) Light availability and seed dispersal
   (v) Zone of influence around individuals (for various processes); for models with x,y coordinates of trees.
4. **Vertical grain –** What is the resolution of the model in the vertical dimension:
   (i) NA (not modeled, i.e. there is no explicit vertical structure in the model)
   (ii) 5 m or coarser
   (iii) 1-5 m
   (iv) <1 m
   (v) continuous.
5. **Vertical extent of crowns –** How large are the modeled tree crowns in the vertical dimension:
   (i) NA (not modeled, i.e. there is no explicit vertical structure in the model)
   (ii) All leaves are at the top of the tree in an infinitesimally small disk)
   (iii) Cylindric crowns
   (iv) More complex crown shape.
6. **Grain of modeled entities –** What biological entities are modeled:
   (i) Size classes (by size, age or height)
   (ii) Cohorts (by age or size; usually these are the same)
   (iii) Individuals.
7. **Life forms considered –** Does the model focus on trees alone:
   (i) Trees only
   (ii) Trees and other life forms (e.g., herb layer).
8. **Focus of application –** What has been the primary objective of model development and application:
   (i) Emphasis on forest structure and composition
   (ii) Emphasis on forest functioning (biogeochemical cycling)
   (iii) Emphasis on forest structure, composition, and functioning.

### b) Growth (GR)

1. **Central state variable –** What is/are the primary state variables in the growth model
   (i.e., those that are calculated first, such as dbh increment in “conventional” gap models, or NPP/biomass in “mechanistic” models, where dbh and h are determined *after* allocation has occurred):
   (i) Age
   (ii) Diameter at breast height (DBH)
   (iii) Height
   (iv) DBH and height
   (v) Bulk tree biomass (lumped)
   (vi) Different tree biomass compartments, e.g. foliage, stemwood, roots, reserves, …
2. **Time step for the update of tree geometry**:
   (i) Decadal
   (ii) Annual
   (iii) Monthly
   (iv) Daily
   (v) Sub-daily.
3. **Time step for modeling of tree productivity**:
   (i) Decadal
   (ii) Annual
   (iii) Monthly
   (iv) Daily
   (v) Sub-daily.
4. **Approach to model productivity & growth**:
   (i) Rule-based
   (ii) An optimum equation of some form, which is reduced by sub-optimal environmental conditions
   (iii) NPP is simulated, which is then distributed to the different plant organs (allocation), no matter what the allocation rules are (cf. item 13, below).
5. **Allocation –** How are the sizes of the different tree compartments/tree dimensions determined:
   (i) NA (no carbon balance, allometric relationships for tree compartments, if any)
   (ii) Fixed coefficients for NPP partitioning (e.g., “20% of NPP go to foliage”)
   (iii) Dynamic coefficients for NPP partitioning (e.g., principle of functional balance etc.).
6. **Height-DBH ratio –** How is the ratio of height to DBH determined:
   (i) NA (not modeled, because at least one of the variables is not considered in the model)
   (ii) Fixed (using an allometric relationship
   (iii) Varies with light availability (no matter how exactly this is done)
   (iv) Varies with nutrient availability (do.)
   (v) Varies with availability of light and nutrients (do.)
7. **Leaf area-DBH ratio –** How is the ratio of leaf area (or leaf biomass/weight) and DBH determined:
   (i) NA (not modeled, because at least one of the variables is not considered in the model)
   (ii) Fixed (using an allometric relationship
   (iii) Varies with light availability (no matter how exactly this is done)
   (iv) Varies with nutrient availability (do.)
   (v) Varies with availability of light and nutrients (do.)
   (vi) Pipe model.
8. **Crown length –** How is the vertical length of the tree crowns determined:
   (i) NA (not modeled, cf. item A.5)
   (ii) Fixed fraction of tree height
   (iii) Depends on light availability.
9. **Crown width –** How is the horizontal extent of the crowns determined:
   (i) NA (not modeled, cf. item A.2)
   (ii) Allometric relationship, fixed
   (iii) Varies dynamically with environmental conditions, e.g. light availability
   (iv) Perfect Plasticity Assumption (PPA).
10. **Crown transparency –** Have the tree crowns a constant transparency, or does this vary by species or the environment:
    (i) NA (light availability across the canopy is not simulated explicitly)
    (ii) Fixed
    (iii) Species-specific
    (iv) Environment-specific
    (v) Species- and environment-specific.
11. **Light extinction across the canopy –** How is light extinction across the canopy calculated:
    (i) Rule-based (e.g., smaller trees are shaded to a fixed extent by larger trees)
    (ii) only vertically
    (iii) some sort of direct beam tracing, simplified
    (iv) synthetic hemispheric photographs (Gap Light Index), full direct beam tracing.
12. **Light response –** How is the growth response to light availability modeled:
    (i) Rule-based
    (ii) Using a simple response curve
    (iii) Using a mechanistic approach based on light use efficiency
    (iv) Using a mechanistic approach based on the Farquhar model.
13. **Environmental influences –** How are the environmental influences combined to affect growth:
    (i) Rule-based
    (ii) Multiplicative approach (as in Botkin et al. 1972)
    (iii) Some other simple approach (e.g. Liebig’s law of the minimum, Geometric mean)
    (iv) A complex, mechanistic approach (e.g. direct impact on PS and R).
14. **Time step for environmental influences –** At what time step are the environmental influences taken into account:
    (i) Decadal
    (ii) Annual
    (iii) Monthly
    (iv) Daily
    (v) Sub-daily.
15. **Temperature –** How is the impact of temperature on tree growth modeled:
    (i) NA (no explicit temperature response in the model)
    (ii) T_min_ in January and T_max_ in July (*applies only to SORTIE, Moran et al. 2021*)
    (iii) Using a response curve, e.g. via the degree-day sum
    (iv) Mechanistic, e.g. temperature dependency of respiration via *Q_10_*.
16. **Soil moisture –** How is the impact of soil moisture on tree growth modeled:
    (i) NA
    (ii) P (precipitation, period to be clarified (*applies only to SORTIE, Moran et al. 2021*)
    (iii) Using a response curve, e.g. via a drought index
    (iv) Mechanistic using stomatal conductance
    (v) Mechanistic, based on some other concept.
17. **Nutrients –** How is the impact of soil-bound nutrients on tree growth modeled:
    (i) NA (not modeled)
    (ii) Using a response curve, e.g. based on nitrogen availability
    (iii) Mechanistic, from a dynamic model of soil C/N/P/… turnover.
18. **CO_2_ –** How is the impact of CO_2_ fertilization on tree growth modeled:
    (i) NA (not modeled)
    (ii) Using a response curve
    (iii) Mechanistic, based on the C_i_/C_a_ ratio.
    (iv) Mechanistic, based on some other concept.
19. **WUE –** How is changing water use efficiency modeled:
    (i) NA (not modeled)
    (ii) Using a response curve
    (iii) Using a mechanistic model (i.e., dynamically modeled ratio of CO_2_ uptake and transpiration).
20. **Crowding –** Is there some factor in the model that represents crowding effects (e.g., competition for space) and/or puts a cap on maximum biomass/basal area/volume:
    (i) NA (not modeled)
    (ii) Rule-based
    (iii) Response curve (irrespective of its form).
21. **Phenology –** How is phenology taken into account:
    (i) NA (not modeled)
    (ii) Very simply, e.g. via degree-days (beginning and end of the growing season)
    (iii) With a simple phenology model, e.g. taking into account winter chilling
    (iv) With a complex phenology model.

### c) Establishment (ES)

1. **Approach –** What process is used for modeling establishment:
   (i) Bernoulli model (random uniform approach; e.g. in JABOWA)
   (ii) Poisson model (producing sapling count data directly; e.g. in FORSKA)
   (iii) Hurdle model (first determine probability, then sapling number; e.g. in FORENA)
2. **Establishment probability –** How is the probability of establishment modeled:
   (i) as a purely random process
   (ii) as a random process modified by environmental factors.
3. **Number of established trees (continuous) –** How is the number of trees to be established determined:
   (i) as a purely random process
   (ii) as a random process modified by environmental factors
   (iii) as a rate (applies only to ED/ED2, probably).
4. **Ingrowth threshold –** What is the size threshold for ingrowth (expressed as h or dbh):
   (i) Age class
   (ii) h < 50 cm
   (iii) 50 < h < 130 cm
   (iv) 0 < dbh < 2 cm
   (v) dbh ≥ 2 cm.
5. **Environmental influences –** How are environmental influences taken into account in the establishment process (general approach):
   (i) binary (as step functions/thresholds)
   (ii) at least partly as continuous variables.
6. **Light –** How is light availability taken into account in establishment:
   (i) NA (not modeled explicitly)
   (ii) Rule-based (e.g., no establishment when shade-intolerant species are in the canopy)
   (iii) via Stand Density Index (SDI)
   (iv) as maximum biomass (when exceeded, no establishment can take place)
   (v) as light availability at the forest floor.
7. **Moisture –** How is soil moisture taken into account in establishment:
   (i) NA (not modeled)
   (ii) Number of wet days during the growing season
   (iii) Minimum AET
   (iv) Soil moisture threshold
   (v) Drought index
   (vi) via NPP.
8. **Temperature –** How is temperature taken into account in establishment:
   (i) NA (not modeled)
   (ii) Degree-days
   (iii) Winter temperature
   (iv) Degree-days and winter temperature
   (v) via NPP.
9. **Frost –** How is frost occurrence (mostly late frosts) taken into account in establishment:
   (i) NA (not modeled)
   (ii) in a simple way (e.g., threshold of a single minimum temperature)
   (iii) with a more complex model (e.g., temperature sequence, pre-conditioning, etc.).
10. **Browsing –** How is browsing by ungulates taken into account in establishment:
    (i) NA (not modeled)
    (ii) static (i.e., same browsing pressure across time, but species-specific effects)
    (iii) dynamic (i.e., browsing pressure varies over time).
11. **Seed production** – How is seed production handled:
    (i) NA (always seeds of all species available)
    (ii) Simple model (e.g. based on age at maturity and fecundity, but no masting)
    (ii) Complex model (including masting).
12. **Dispersal –** How is seed dispersal taken into account in establishment:
    (i) NA (not modeled, i.e. assumption of unlimited seed availability)
    (ii) Rule-based (e.g. 50% of seeds land on the nearest neighbor patches)
    (iii) Single exponential dispersal kernel
    (iv) Double exponential dispersal kernel
    (v) More complicated approach.
13. **Vegetative reproduction –** How is vegetative reproduction taken into account in establishment:
    (i) NA (not modeled)
    (ii) Sprouting
    (iii) Root suckers
    (iv) Sprouting and root suckers.

### d) Mortality (MO)

1. **Background mortality: species- or PFT-specific? –** Is the background mortality formulation general, species-specific, or PFT-specific?
   (i) NA (not modeled)
   (ii) Unspecific (same probability for all individuals)
   (iii) Species-specific
   (iv) PFT-specific (even for models that resolve species, mortality may refer to functional groups).
2. **Background mortality formulation –** What is the functional form of the background mortality formulation:
   (i) NA (not modeled)
   (ii) Death occurs when trees reach their maximum longevity
   (iii) Constant over age or size (e.g., so-called “maximum age-dependent”)
   (iv) Increasing with age
   (v) Increasing with size (e.g., dbh)
   (vi) Increasing with size and age.
3. **Stress-related mortality –** How is enhanced stress/low vitality impacting mortality?
   (i) NA (not modeled)
   (ii) constant
   (iii) via dbh increment
   (iv) via growth efficiency
   (v) via resource depletion (reserves)
   (vi) productivity-related.
4. **Disturbance mortality –** Is disturbance mortality in a general sense included in the model, and if so, how?
   (i) NA (not modeled)
   (ii) using a very simple approach, e.g. general disturbance probability that kills all trees
   (iii) using a more complex approach.
5. **Windthrow –** Is windthrow included, and how is it modeled:
   (i) NA (not modeled)
   (ii) Empirical approach, relatively simple (e.g., risk related to tree height and species alone)
   (iii) Mechanistic approach, more complicated (e.g., risk related to tree height, species, local environment, etc.)
6. **Bark beetles –** Are bark beetle dynamics included, and how are they modeled:
   (i) NA (not modeled)
   (ii) Empirical approach, relatively simple (e.g., risk related to temperature [beetle pressure] and drought [tree susceptibility])
   (iii) Mechanistic approach, more complicated.
7. **Fire –** Is fire included, and how is it modeled:
   (i) NA (not modeled)
   (ii) Empirical approach, relatively simple (e.g., fire spread is modeled implicitly using terrain and drought variables alone)
   (iii) Mechanistic approach, more complicated (e.g., fire spread is modeled explicitly as a function of terrain, wind speed, fuel load, …)

### e) Soil moisture (SM)

1. **Vertical resolution –** What is the vertical resolution of the soil moisture model:
   (i) NA (e.g., models that work with precipitation directly)
   (ii) One layer
   (iii) Two layers
   (iv) Multiple layers.
2. **Temporal resolution –** What is the temporal resolution (grain) of the soil moisture model:
   (i) NA (same as above)
   (ii) Annual
   (iii) Monthly
   (iv) Daily
   (v) Sub-daily.
3. **Drought –** How are soil moisture dynamics *linked* to tree growth and demography:
   (i) NA (same as above)
   (ii) Number of dry days during the growing season (i.e., days with soil moisture below permanent wilting point or another soil moisture threshold)
   (iii) AET
   (iv) via an approach relying on the ratio of AET to PET (in some way)
   (v) via soil moisture directly
   (vi) via stomatal conductance or some other physiology-based concept.
