# Supplementary material for: The evolution, complexity and diversity of models of long‐term forest dynamics
Source: J Ecol. 2022 Sep 8;110(10):2288–307. doi: 10.1111/1365-2745.13989 (PMC9826524; doi:10.1111/1365-2745.13989)
Supplement: Supplementary file 1 — Appendix S1 [file JEC-110-2288-s001.zip › JEC_13989_Genealogy Paper SM2&3 (tables & figures).docx]

## SM 2: Supplementary Tables

Tab. S1: a) Average complexity of the attributes of the respective process group by model type. b) Diversity (expressed as the standard deviation) of the complexity of the attributes of the respective group. Green, orange and red shading of the cells indicates highest, intermediate, and lowest complexity or diversity per attribute group, respectively.

| a) | Stand models | Landscape models | Global models |
| --- | --- | --- | --- |
| BA | -0.01 | -0.19 | 0.24 |
| GR | -0.13 | -0.40 | 0.70 |
| ES | 0.02 | 0.29 | -0.37 |
| MO | -0.30 | 0.54 | -0.06 |
| SM | -0.13 | -0.56 | 0.88 |
|  | | | |
| b) | Stand models | Landscape models | Global models |
| BA | 0.92 | 1.22 | 0.56 |
| GR | 0.71 | 1.12 | 0.50 |
| ES | 0.72 | 0.86 | 0.71 |
| MO | 0.70 | 1.11 | 0.47 |
| SM | 0.87 | 1.14 | 0.27 |

## SM 3: Supplementary Figures


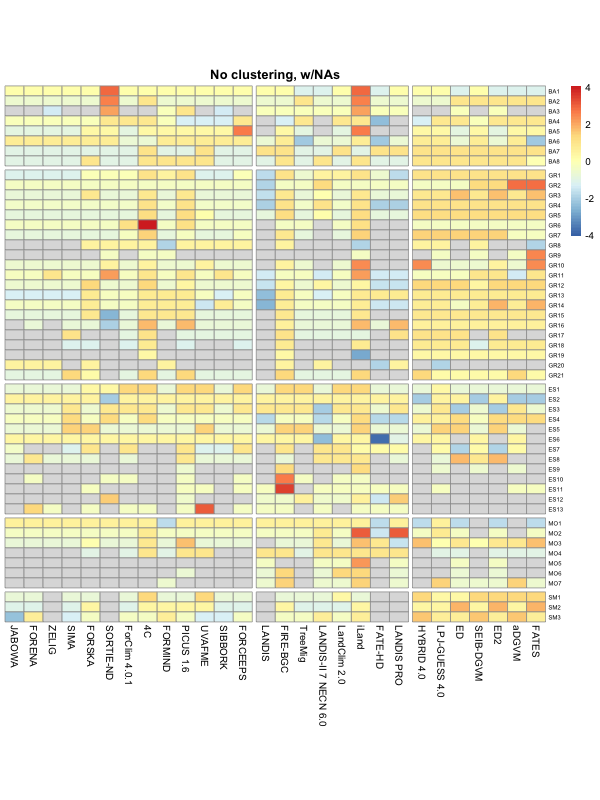


**Complexity**

Fig. S1: Heat map of the 28 models arranged according to stand, landscape, and global models (from left to right, and within each model category ranked according to first published documentation). Gaps between rows indicate boundaries between the process groups (BA, GR, ES, MO, and SM). Numbers to the right of the rows indicate the respective attribute number (cf. SM 1). Grey cells indicate attributes that are not considered in a model.


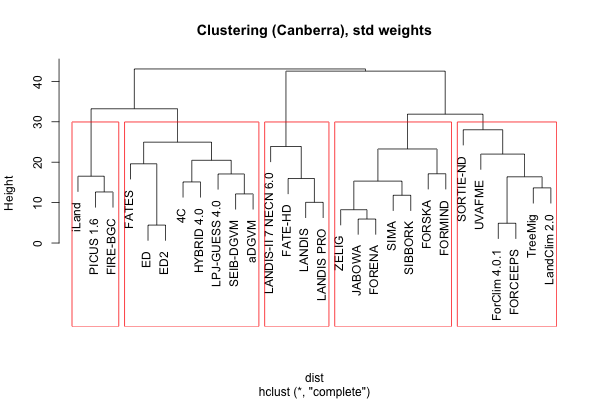


Fig. S2: Hierarchical clustering of the 28 models using Canberra distances and the ‘complete’ clustering method.

Fig. S3: Matrix of similarities between the 28 models (defined as 1 – Canberra distance), scaled between 0 (minimum similarity) and 1 (maximum similarity). Underlying horizontal bars are proportional to the similarity measure and are shown for easier orientation.
